# Heavy metals mitigation and growth promoting effect of endophytic *Agrococcus terreus* (MW 979614) in maize plants under zinc and nickel contaminated soil

**DOI:** 10.3389/fmicb.2023.1255921

**Published:** 2023-11-09

**Authors:** Asim Shahzad, Anam Siddique, Shazia Ferdous, Muhammad Ahmar Amin, Mingzhou Qin, Uzma Aslam, Muhammad Naeem, Tasmia Bashir, Abdul Shakoor

**Affiliations:** ^1^The College of Geography and Environment, Henan University, Kaifeng, China; ^2^Department of Botany, Mohi-Ud-Din Islamic University, AJ&K, Pakistan; ^3^Rawalpindi Women University, Rawalpindi, Pakistan; ^4^Department of Plant Science, School of Agriculture and Biology, Shanghai Jiao Tong University, Shanghai, China; ^5^Department of Botany, Rawalpindi Women University Rawalpindi, Rawalpindi, Pakistan

**Keywords:** endophytic bacterial, *Agrococus terreus*, *Zea mays*, heavy metals stress, zinc contamination nickel contamination, antioxidant enzymes

## Abstract

**Introduction:**

Heavy metals such as iron, copper, manganese, cobalt, silver, zinc, nickel, and arsenic have accumulated in soils for a long time due to the dumping of industrial waste and sewage. Various techniques have been adapted to overcome metal toxicity in agricultural land but utilizing a biological application using potential microorganisms in heavy metals contaminated soil may be a successful approach to decontaminate heavy metals soil. Therefore, the current study aimed to isolate endophytic bacteria from a medicinal plant (*Viburnum grandiflorum*) and to investigate the growth-promoting and heavy metal detoxification potential of the isolated endophytic bacteria Agrococus tereus (GenBank accession number MW 979614) under nickel and zinc contamination.

**Methods:**

Zinc sulfate and nickel sulfate solutions were prepared at the rate of 100 mg/kg and 50 mg/kg in sterilized distilled water. The experiment was conducted using a completely random design (CRD) with three replicates for each treatment.

**Results and Discussion:**

Inoculation of seeds with *A. tereus* significantly increased the plant growth, nutrient uptake, and defense system. Treatment T4 (inoculated seeds), T5 (inoculated seeds + Zn100 mg/kg), and T6 (inoculated seeds + Ni 100 mg/kg) were effective, but T5 (inoculated seeds + Zn100 mg/kg) was the most pronounced and increased shoot length, root length, leaf width, plant height, fresh weight, moisture content, and proline by 49%, 38%, 89%, 31%, 113%, and 146%, respectively. Moreover the antioxidant enzymes peroxidase and super oxidase dismutase were accelerated by 211 and 68% in contaminated soil when plants were inoculated by *A. tereus* respectively. Similarly the inoculation of *A. tereus* also enhanced maize plants’ absorption of Cu, Mn, Ni, Na, Cr, Fe, Ca, Mg, and K significantly. Results of the findings concluded that 100 mg/kg of Zn and Ni were toxic to maize growth, but seed inoculation with *A. tereus* helped the plants significantly in reducing zinc and nickel stress. The *A. tereus* strain may be employed as a potential strain for the detoxification of heavy metals

## 1. Introduction

Food security necessitates; an increase in global food production. The primary emphasis of agricultural studies is on crop cultivation. These analyzes overlooked the variability and unreliability of the annual grain output- as well as the inherent instability of production (Knapp and Van Der Heijden, 2018). Research on foods that may contain heavy metals has been prompted by increasing emphasis on food safety (Adamson et al., 2018). Pollution of farmlands with hazardous metals is a major ecological threat. These compounds are widespread and have negative short- and long-term effects on plant development, making them prime examples of soil pollutants (Zhang et al., 2023). All living forms are endangered by heavy metals in the soil (Ding et al., 2022). Heavy metals are chemically metallic elements with relatively high density and low toxicity. Nevertheless, this physical characteristic is very harmful to plants and other creatures (multiple metabolic systems may be disrupted by the toxicity of heavy metals. Regardless of the exact symptoms and signs associated with each heavy metal, even minimal exposure to these compounds has a negative influence on plant growth (Dutta et al., 2018). Multiple pathways exist for the transport of heavy metals, which may have devastating health consequences (Balali-Mood et al., 2021).

Oxidative stress and heavy metal toxicity pose severe threats to agricultural systems (Hou et al., 2020). In addition to other environmental pressures, the presence of hazardous metals causes plants to increase their enzymatic defense system (Haider et al., 2021). By replacing metal ions with metal-enzymes, these heavy metals inhibit a range of plant activities, including fertilization, as well as a number of plant morphological changes (Morkunas et al., 2018), Alterations in the physiological and biochemical cycles of plants reduce plant growth (Ali et al., 2020). Metals never degrade; however, when their concentrations in a facility surpass safe levels, they have a negative impact on plant health and the environment. Inhibition of cytoplasmic molecules and breakdown of the cell structure due to oxidative pressure are two direct detrimental effects of excessive metal concentrations (Mishra et al., 2017). Metals that may be absorbed by plants are dissolved in the soil solution or expelled by roots (Sarwar et al., 2017). Even when plants require certain heavy metals for development and sustenance, excessive amounts of these elements may be harmful to plant life.

Inhibition of cytoplasmic enzymes and oxidative damage to the cellular structure are direct adverse consequences at higher dosages (Arif et al., 2016) and the activities of soil microorganisms and the detrimental impacts of heavy metals may also inhibit plant development and growth. For example, owing to the high concentration of minerals, the breakdown of organic matter in the soil is hindered, and the number of beneficial bacteria decreases, causing a loss of nutrients in the soil (Urra et al., 2019). As a consequence of heavy metal interference with the function of soil microorganisms, the activity of enzymes crucial to plant metabolism is also inhibited. These detrimental effects result in stunted plant development, which may ultimately lead to plant death (Shi et al., 2022). Another unfavorable consequence of heavy metal accumulation is the release of reactive oxygen species (ROS), the composition of which is determined by the equilibrium between ROS production and ROS elimination in plants, which is influenced by factors such as the presence of heavy metals, temperature, light intensity, etc (Zandi and Schnug, 2022). Carbon dioxide (CO_2_) fixation in chloroplasts contributes considerably to ROS formation owing to the severe reduction in the photosynthetic electron transport chain (Sachdev et al., 2021). Highly reactive oxygen species (ROS) may interact with macromolecules such as deoxyribonucleic acid, oils, enzymes, and lipids, as well as other important biological components such as chemical interactions within the cellular system and redox potential (De Almeida et al., 2022). Heavy metal ions, such as Copper (Cu), zinc (Zn), manganese (Mn), and iron (Fe), are important for plant metabolism, however, their excess is very toxic. For example, when Zn and Mn are abundant, they impact plant development and reduce the effects of Fe (Ferrol et al., 2016). Maize (*Zea mays* L.), which ranks third among cereals after wheat and rice and serves as a staple food, is a major cereal crop grown worldwide as a food and feed crop (Chen et al., 2022). Maize, is naturally rich in carbohydrate, which is consumed as a major food source in developing countries, However because of the contamination of soil with heavy metals, the accumulation of heavy metals in maize plants reaches beyond the permissible limits (Vongdala et al., 2019) and these heavy metals taken up by maize plants, alter their physiological features, and molecular and cellular attributes even at low concentrations in soil (Rizvi et al., 2022).

By using a biological concentration of microorganisms, contaminated soil can be successfully decontaminated from heavy metals (Anastopoulos et al., 2019). Bacteria, Firmicutes, and Actinobacteria have been proposed as alternatives for the decontamination of metal-polluted soils with high concentrations of Mn, Pb, and As (Xiao, 2017). Bacteria are vital microorganisms for the removal of heavy metal-contaminated soils (Bacteria have the ability to reduce and detoxify pollutants such as heavy metals in soil by the process of immobilization, activation, absorption, and transformation; Hassan et al., 2017). The presence of free-living and specific plant growth promoting rhizobacteria (PGPR) in the rhizosphere soil significantly helps plant root development and plant survival by altering the availability of nutrients, thereby increasing plant growth (Nadeem et al., 2014). At different stages of a plant’s life, all its tissues interact with microorganisms; nonetheless, this interaction seldom causes harm to the plant. Bacteria are found in the roots and surfaces of plants. Plants emit chemicals and nutrients that maintain and attract microbes (Rajkumar and Kurinjimalar, 2021). Microorganisms produce compounds that promote plant development (Schirawski and Perlin, 2018). The primary carbon source for soil bacteria is carbon from photosynthesis or plant residue (Wang et al., 2020).

Endophytic microorganisms and plants develop symbiotic interactions because both parties gain from these interactions. The host plant absorbs compounds that boost nutrient absorption and growth in return for room and protection (Riaz et al., 2021). Roots often contain more bacteria than dirt. This indicates that plant roots reject 5–30% of the microscopic organic acids, amino acid, and carbohydrate molecules that bacteria require for survival (Liu et al., 2017). Endophtic bacteria can penetrate plant parts such as roots, flowers, leaves, and branches. These microorganisms may have developed in the seeds (Halwas, 2017). Endophytic bacteria were isolated from several monocot, dicot, woody, and herbaceous plants, including oak (*Quercus* L.), pear (*Pyrus* L.), sugar beet (*Beta vulgaris* L.), and corn (*Zea mays* L.) (Chebotar et al., 2015). Among the endophytic bacteria of maize are *Pantoea*, *Rhanella*, *Rhizobium*, *Herbaspirillum*, *Pseudomonas*, *Brevundimonas*, *Enterobacter*, and *Burkholderia*. *Pseudomonas fluorescence* produces a siderophores and phosphate solubilizers. *Vibernum grandiflorum* is a medicinal plant that includes approximately 230 species of deciduous Adoxaceae shrubs. Most members of the genus *Viburnum* are indigenous to tropical and temperate regions. Pakistan is home to six species of *Viburnum*, including *Viburnum contonifolium*, *Viburnum tinus*, *Viburnum cylindricum*, and *Viburnum grandiflorum*. *Viburnum* root and stem extracts exhibit antifungal, insecticidal, and phototoxic effects (Raoof and Siddiqui, 2013). It is used in traditional medicine as a blood cleanser, laxative, and a diuretic. It is calming and antispasmodic, and helps in liver conditions. Its branches are utilized to create toothbrush bristles. *Viburnum* branches are both a source of fencing materials and fuel (Kumar et al., 2020). Information on endophytic bacteria from medicinal plants and their use as plant promoters is rare, and a detailed study is required on the *Agrococcus terreus* strain, on which very few reports are available to date; therefore, the current study was designed to isolate the bacterial endophytes from the roots of a medicinal plant, *Viburnum grandiflorum*, and to investigate the growth-promoting potential of isolated endophytic bacteria (*Agrococcus terreus*) in maize plants grown in nickel- and Zn-contaminated soil.

## 2. Methodology

### 2.1. Sample collection

**Figure 1:**
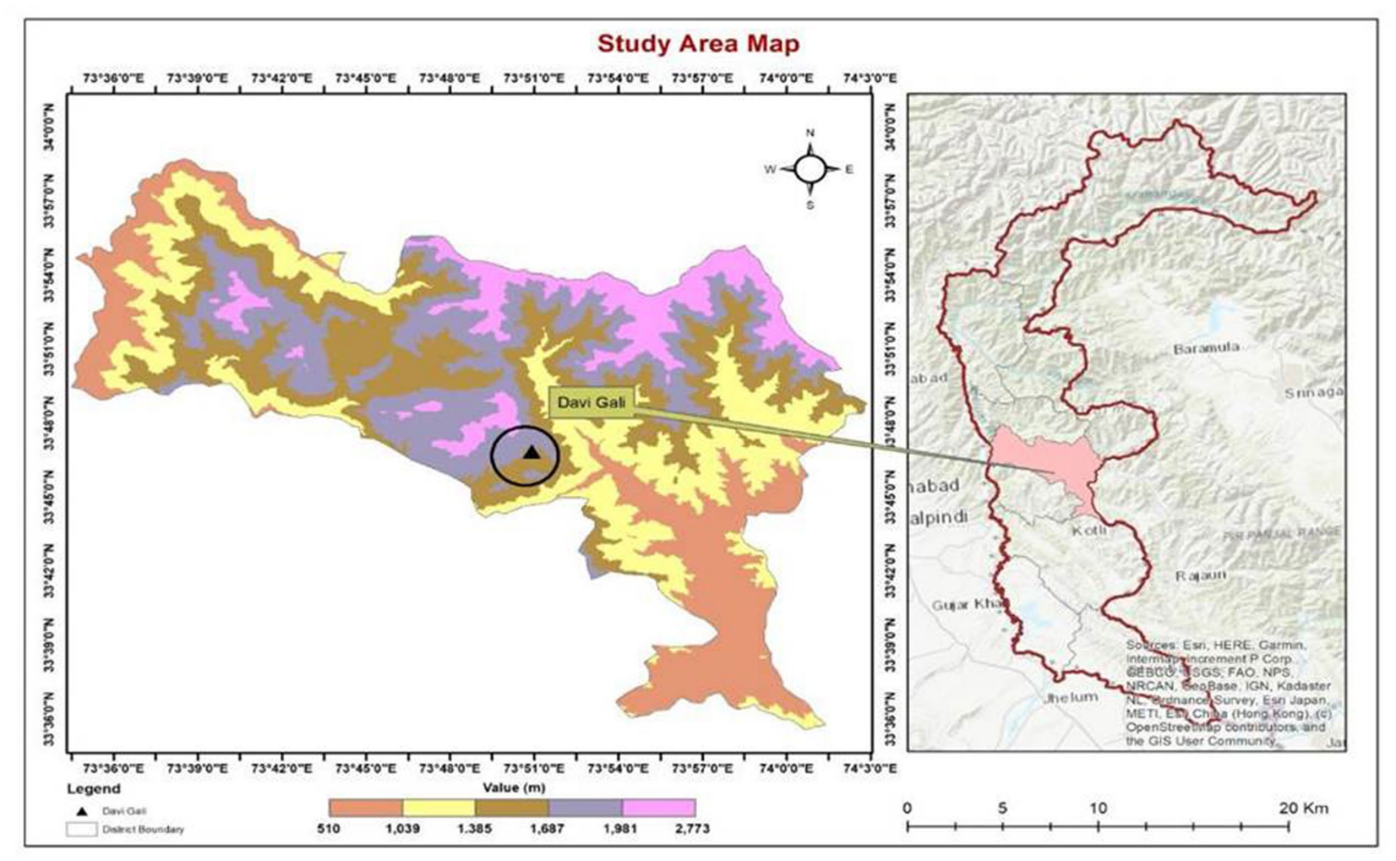
Study area map.

The roots of *V. grandiflorum* were collected from District Poonch (Davi Gali) at an elevation of 5,000 ft. Healthy and mature plants were selected and collected for the isolation of bacteria. The roots of the selected plant *Viburnum grandiflorum* were carefully cut and packed in zip–lock plastic bags. The samples were then brought to the laboratory for further research (Figure 1).

### 2.2. Isolation of bacterial endophytes

Bacterial endophytes were isolated from the roots of *Viburnum grandiflorum* in nutrient agar medium using the method described by Rekha et al. (2015). Small pieces of root were made using a sterilized knife. Surface sterilization was performed by sequential cleaning with ethanol (70%) for 5 min, 1% HgCl2 for 1–2 min and double-distilled water 5–6 times for 2–5 min. Sterile root pieces were macerated in phosphate buffer (PB) at PH 7.0.After maceration, the samples were grinded with a disinfected pestle and mortar in 9.5 mL of the final buffer wash with distilled water grinded. From grinded sample 10 mL was taken into falcon tube. Two replicate samples were prepared and centrifuged at 3000 rpm for 15 min. Afloat was collected, and serial dilutions from this extract were prepared in phosphate buffer (10–5, 10–6 and 10–7). From each dilution a 0.1 mL extract was inoculated on separate Petri plates containing nutrient agar media. The plates were incubated at 37C° observation was taken after 48 to 72 h. Bacterial isolates were picked from the plates and purified using streaking techniques. This isolation process was repeated until pure colonies were obtained. Based on morphology and color, different colonies were obtained. Gram staining was performed to identify Gram-positive and Gram-negative bacteria (Rekha et al., 2015).

### 2.3. Identification, and 16S rRNA sequencing of endophytic bacteria

Genomic DNA was extracted from the bacterial strains using the Bacterial Genomic DNA Kit (Smith et al., 2003). The bacterial strain was grown in nutrient broth and incubated overnight to extract the genomic DNA. About 1.5 mL of saturated bacterial culture was transferred to an Eppendorf tube and harvested by centrifugation at 10000 rpm for 2 min. The pellets were collected and harvested again with 1.5 mL of cell culture. Thereafter, the pellets were drained using a paper towel. The pellets were re-suspended and lysed in 450ul μL transcription buffer (TF) with dynamic pipetting. To remove proteins and cell remains 45ul of 10% sodium dodecyl sulfate (SDS) solution and 5ul of 20 mg/mL proteinase K were added, mixed well, and incubated for an hour at 37oC. Thereafter, the clear supernatant was transferred to a clean vial and approximately 500ul phenol-chloroform was added and mixed energetically by inverting the tube until the phases were completely mixed. The vial was centrifuged at10000rpm for 2 min. The upper aqueous phase was transferred to a new Eppendorf tube, mixed with a phenol-chloroform mixture again, and centrifuged at 10000 rpm for 5 min. The obtained aqueous phase was transferred to a new tube and 50ul of sodium acetate was added and mixed well by hand. Thereafter, 300ul of isopropanol was added and mixed gently to precipitate the DNA, which was centrifuged at 10000 rpm for 2–5 min. The DNA was washed with 1 mL of 70% ethanol for 30 s and centrifuged. DNA was drained with ethanol, dried, and re-suspended in 50-100ul. T.E buffer and DNA were stored at -20oC. The similarity index of the sequences of the isolated endophytic bacterial strains was determined using BLAST in the online tool NCBI nucleotide BLAST. By using sequence match application and BLAST to show the resemblance of new sequences with the reference sequences in the databases (Auld et al., 2013). The phylogenetic association of the isolated bacterial strains was resolved using the neighbor-joining technique (Saitou and Nei, 1987). A phylogenetic tree was constructed using molecular evolutionary genetics (Stefanova et al., 2015).

### 2.4. Culturing of *Agorcocus tereus*

The culture of the isolated *A. tereus* bacteria was identified as (Acc. No. MW 979614) was spread over nutrient agar medium. Nutrient agar was prepared by dissolving 28 gm/1000 mL of nutrient agar medium in distilled water.

### 2.5. Preparation of inoculums

For inoculum preparation, 250 mL nutrient broth medium was sterilized. The medium was autoclaved for 45 min at 121°C. The broth was inoculated with a 24 h old culture and incubated at 32°C and 150 rpm in a shaker incubator (NB-205LF) for 3–5 days.

### 2.6. Seed inoculation

Maize (*Zea mays*) seeds were obtained from the National Agricultural Research Center Islamabad (Pakistan). The surface of the seeds was sterilized by washing with 95% ethanol and then immersing them in 10% Clorox for 2–3 min. The seeds were then washed in sterile filtered H_2_O 2–3 times. Seeds were gently stirred to remove any loose dirt collected from seedlings (Omid et al., 2010). Plant height, shoot length, root length, leaf length, number of leaves, fresh plant weight, moisture content, leaf proline, leaf protein, leaf sugar estimate, peroxidase dismutase (POD), and super oxide dismutase (SOD) were measured and analyzed (Liu et al., 2010).

### 2.7. Preparation of zinc and nickle solution and treatment application

Analytical grade zinc sulfate and nickel sulfate were used during this study, and Ni and Zn solutions were prepared for different treatments in autoclaved distilled water. Approximately 100 mg of zinc sulfate and nickel sulfate were dissolved in 100 mL of water. Both zinc and nickel solutions were added to 1 kg of soil (100 mg/100 mL/ 1 kg) (Ye et al., 2017). Eight different treatments were used (Table 1), and the plants were harvested after 28 days of germination.

**Table 1:** Treatments made in experiment.

Treatments	Conditions
T1	Control (No Heavy Metals)
T2	Seed + Zinc (Zn)100 mg/kg
T3	Seed + Nickel (Ni)100 mg/kg
T4	Inoculated seeds
T5	Inoculated Seed +Zn100mg/kg
T6	Inoculated Seed +Ni100mg/kg
T7	Inoculated Seed + Ni 50 mg/kg + Zn 50 mg/kg
T8	Seed +Ni50mg/kg + Zn50mg/kg

### 2.8. Plant parameters studied

Root and shoot lengths were measured in centimeters from the apex to the tip of the shoot and root, respectively (Prajapati et al., 2008). The seedlings were removed from their containers, the excess dirt was washed off, and the final weight in grams was determined (Banerjee et al., 2016). To determine the dry weight, the seedling roots and shoots were oven-dried overnight at 70 0C, and the weight was measured in grams (Ricigliano, 2015).

### 2.9. Plant nutrient analysis

The per-chloric acid digestion method was used to assess the nutrient content of the maize plants (McLaren et al., 2012). This technique was used to determine the presence of nutrients in plant components. Plant leaf material (0.25 g) was used for the nutrient analysis.

Cations in plants = (ppm in extract - blank) × A × dilution factor W A = Total volume of extract (mL) W=Weight of the dry plant.

### 2.10. Leaf protein contents

The protein extract of fresh maize plant leaves was tested using the most effective technique (Rahu et al., 2021). About 0.1 g and fresh maize leaves were used to determine the protein content. Maize plant leaves (0.1 g) were placed in a phosphate buffer. The mixture was centrifuged at 3000 rpm for 10 min. The supernatant was mixed with purified water, and the volume was increased to 1 mL. The resulting solution was added to 1 mL of alkaline CuSO4 reagent and shaken for 10 min, after which folline reagent was added to the solution and incubated for 30 min at 28 ± 2°C. The absorbance was measured at 650 nm using a spectrophotometer.

### 2.11. Leaf proline content

The proline content of the maize leaf extracts was evaluated (Sairam and Srivastava, 2000). About 0.1 g of fresh maize plant leaves were used to examine proline content. Maize plant leaves (0.1 g) were placed in 10 mL of methanol. After that the mixture was centrifuged at 3000 rpm for 10 min. After 24 h, the mixture was refrigerated. After 24 h, the mixture was again centrifuged.1 mL and 5% phenol was added and incubated at room temperature for at least 1 h. Then, sulfuric acid (2.5 mL of sulfuric was added and the absorbance was measured at 490 nm.


$$\mathrm{Proline}=K^{*}\mathrm{Dilution}\;{\mathrm{Factor}}^{*}\mathrm{Optical}\;\mathrm{density}/\mathrm{weight}\;\mathrm{of}\;\mathrm{sample}$$

The K value is 9.6.

### 2.12. Leaf sugar content estimation

Fresh maize leaves (0.5 g) were homogenized in 10 mL distilled water. The extract was centrifuged at 3000 rpm for 5 min. The supernatant was collected and incubated for 1 h at room temperature with 1 mL of 80% phenol. Following the incubation period, 5 mL sulfuric acid was added. The samples were incubated for 4 h at room temperature. Readings were recorded at 420 nm. The sugar value was determined using the given formula.


$$\begin{array}{l} \mathrm{Total}\;\mathrm{sugar}\;\left(\mathrm{\mu g}/g\right)=\\ \frac{k\;\mathrm{value}\;\mathrm{of}\;\mathrm{sugar}*\mathrm{Dilution}\;\mathrm{factor}*\mathrm{absorbance}\;\mathrm{value}\;\left(\mathrm{mg}\right)}{\mathrm{Weight}\;\mathrm{of}\;\mathrm{sample}\;\left(g\right)} \end{array}$$

### 2.13. Peroxidase (POD) analysis

The peroxidase dismutase activity of maize plant leaves was investigated using a standard procedure (Van Assche et al., 1988). Approximately 1 g of cold maize leaves was used to examine the POD antioxidant enzyme.1 g of frozen maize leaves was placed in an icy mortar with 0.5 M calcium chloride solution. The extract was centrifuged at 1000 rpm for 10 min, and the supernatant was transferred to clean test tubes and stored in a freezer. The pellet of the cell wall extract remaining in the centrifuge tube was 2.5 mL of ice-cold mixed calcium chloride solution 0.5 M and centrifuged.

The supernatants were collected twice.

The reaction mixture was prepared as follows:

0.1 mL extract+1.5mlMES + 0.5 mL p-Phenylenediamine+0.45mlHydrogen peroxide.

Add MES Buffer in blank cuvette. At 510 nm the reading was recorded. In two times The readings were recorded twice, at 0 min and 3 min.

### 2.14. Superoxide dismutase analysis

The superoxide dismutase assay for maize plant tissue was assessed (Mushtaq et al., 2022) Mix (a) about 0.2 g of frozen maize leaves were used to examined the SOD enzyme. Plant tissue (b) 0.2 g of plant tissue was placed in 1 g polyvinylpyrrolidone (PVP polyvinylpyrolidone) +0.0278gNaEDTA solutions in a cooled pestle and mortar. Mix (c): The mixture was centrifuged at 4°C for 10 min. The supernatant was collected and raised up to 8 mL by adding phosphate buffer of pH 7.(Mix d) 0.0278 g NaEDTA +1.5 g Methionine +0.04 g Nitro blue tetrazolium chloride (NBT) was dissolved in 100 mL of phosphate buffer (pH 7.8).(Mix e)Take 10 mL from mix (d) and raise its volume up to 50 mL with pH 7.8 Phosphate buffer.(Mix f)Dissolve the 0.0013 g of riboflavin in 100 mL of phosphate buffer (pH 7.8).(Mix g)Take 20 mL from mix (f) and raise its volume to 50 mL with distilled water.

Three types of assays were performed: 1st Reference, 2nd Blank and 3rd Reaction mixture.

The reference sample was mixed well and kept in the dark, and the reaction mixture was kept in the light chamber for approximately 20 min. Absorbance was measured at 560 nm using a spectrophotometer.

### 2.15. Statistical analysis

The study was carried out in pots using a complete randomized design (CRD) with Statistix 8.1. The data for various parameters were obtained after 28 d of seedling growth. Triplicate values were recorded for each treatment (Zhang et al., 2021).

## 3. Results

The present investigation was performed for the isolation and Characterization of endophytic bacteria from the roots of *Viburnum grandiflorum* (guch) and its application on maize plant. Total of four bacterial colonies were isolated from the roots of *Viburnum grandiflorum*.

### 3.1. Identification and 16 s rRNA sequencing of endophytic bacteria

Two endophytic bacterial strains were isolated from *Viburnum grandiflorum* out of which one bacterial strain 16S rRNA sequence showed 93% similarity with *Agrococcus terreus*.

### 3.2. Phylogenetic relation of the isolated bacteria from *Viburnum grandiflorum*

**Figure 2:**
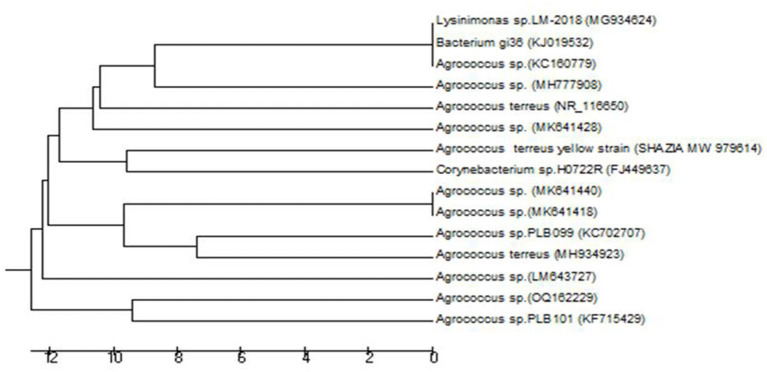
Neighbor–Joining Tree of *Agrococcus terreus* Isolated from *Viburnum grandiflorum*. The evolutionary history was inferred using the UPGMA method [1]. The optimal tree with the sum of branch length = 126.99494267 is shown. The tree is drawn to scale, with branch lengths in the same units as those of the evolutionary distances used to infer the phylogenetic tree. The evolutionary distances were computed using the Maximum Composite Likelihood method [2] and are in the units of the number of base substitutions per site. The analysis involved 15 nucleotide sequences. Codon positions included were 1st + 2nd + 3rd + Noncoding. All positions containing gaps and missing data were eliminated. There were a total of 706 positions in the final dataset. Evolutionary analyzes were conducted in MEGA6 [3].

Figure 2 shows the phylogenetic relationship of the strain isolated from *Viburnum grandiflorum*. The isolated *A. terreus* strain branched off from a group of clades. The *A. terreus* group during evolution did not show much similarity with any clades, but with the ancestor of the clades. There were 10 positions in the final dataset. In the first clade, one species from France was closely related to a species from India, and these two species were next related to Canadian species. In the next clade, one species from Sweden was closely related to the species from China. In the next clade, one species from Egypt was closely related to the species from Switzerland, and these two species were closely related to species from China.

### 3.3. Effect of zinc (Zn) and nickel (Ni) on plant parameters maize seeds germination %

**Figure 3:**
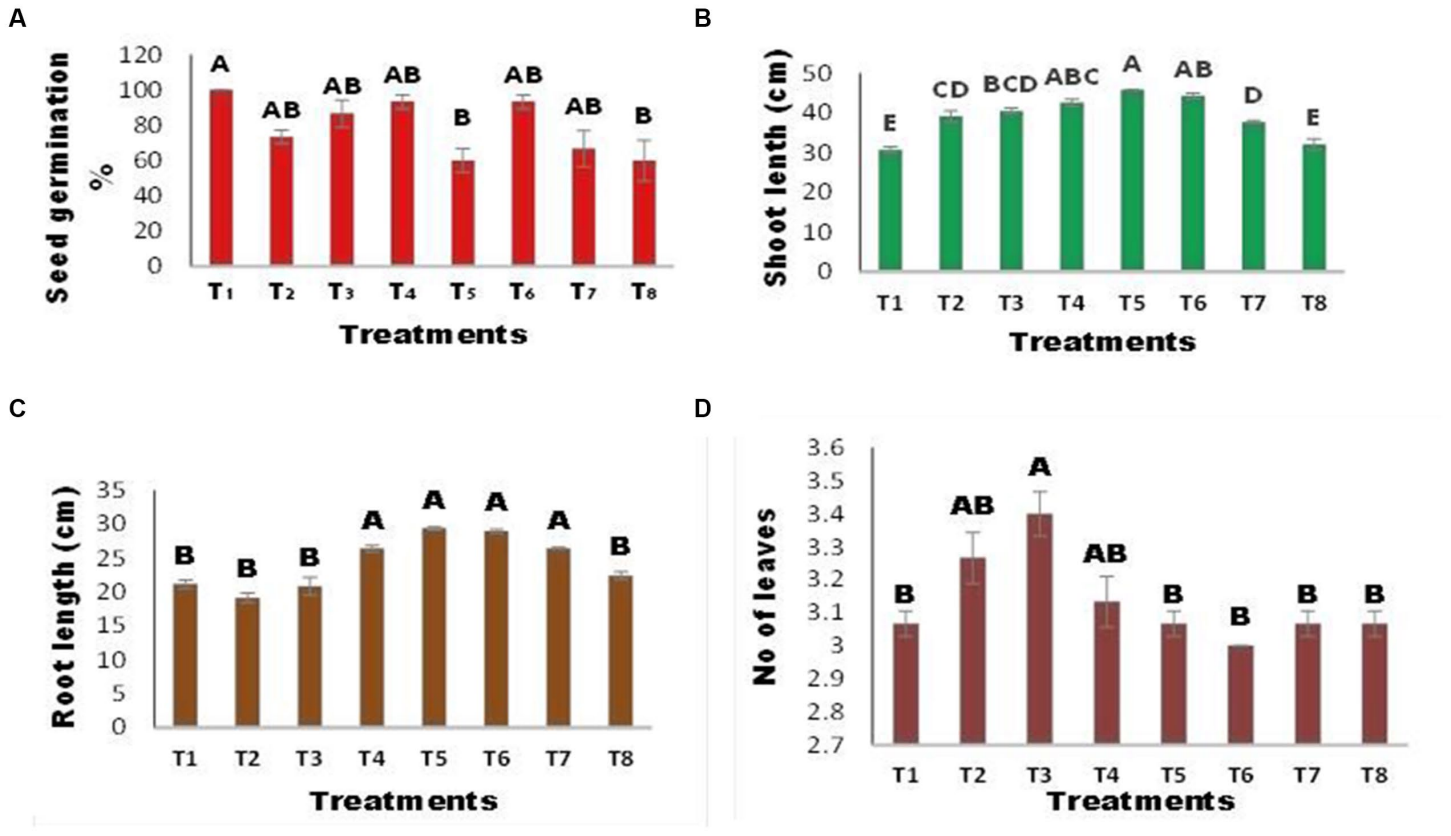
Effect of Zinc (Zn) and Nickel (Ni) on **(A)** Germination % **(B)** Maize Shoot Length **(C)** Root length **(D)** No of leaves The bars sharing common letter are non-significantly different otherwise vary significantly at *p* > 0.05. T1 = control, T2 = seed+Zn100mg/kg, T3 = seed+Ni100mg/kg, T4 = inoculated seed, T5 = inoculated seed+Zn100mg/kg, T6 = inoculated seed+Ni100mg/kg, T7 = inoculated seed+Ni 50 mg/kg + Zn50mg/kg, T8 = seed+Ni 50 mg/kg + Zn 50 mg/kg.

**Figure 4:**
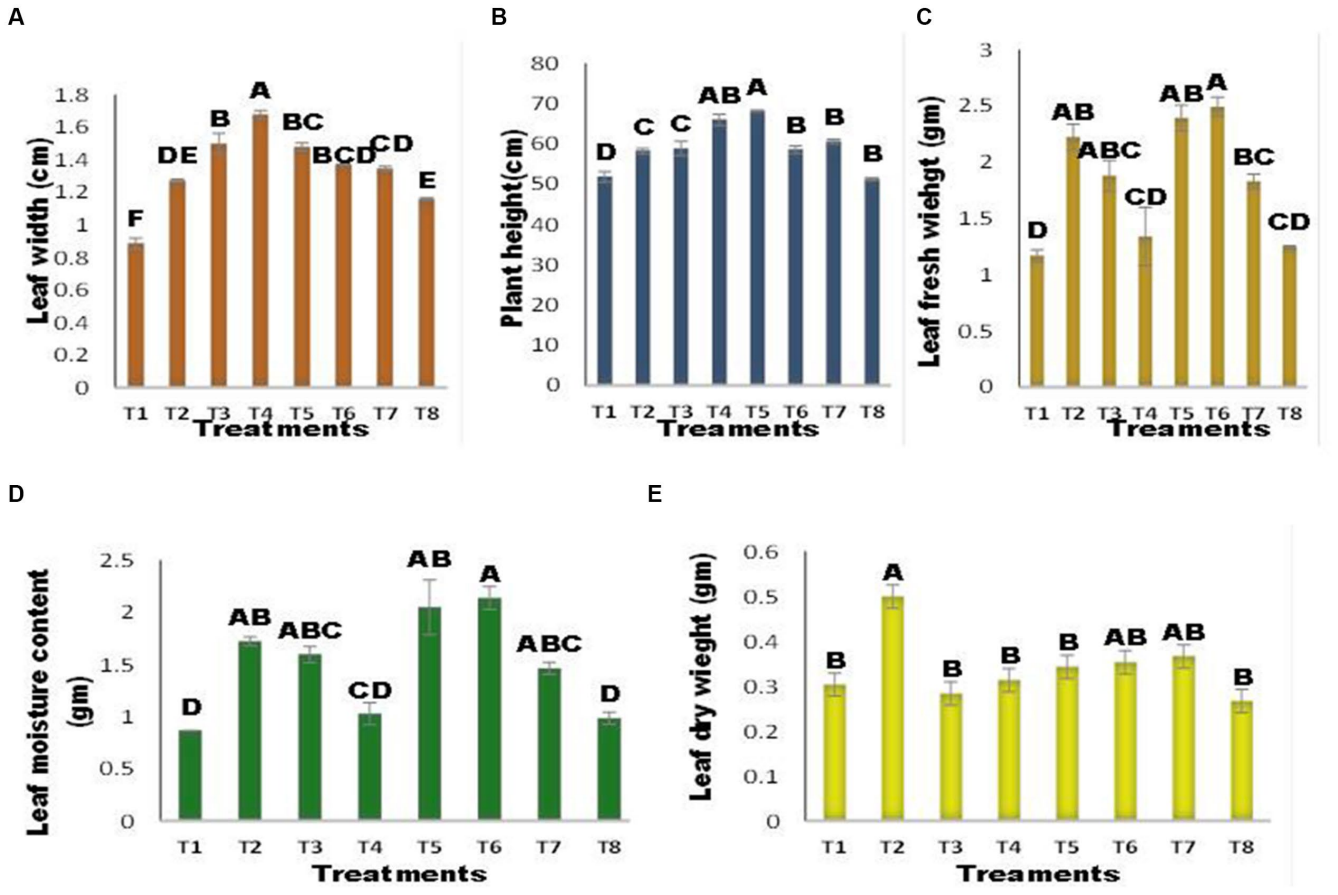
Effect of Zinc (Zn) and Nickel (Ni) on **(A)** Leaf width**(B)** Plant height **(C)** Leaf fresh weight **(D)** Leaf Moisture content **(E)** Leaf dry weight. The bars sharing common letter are non-significantly different otherwise vary significantly at p > 0.05. T1 = control, T2 = seed+Zn100mg/kg, T3 = seed+Ni100mg/kg, T4 = inoculated seed, T5 = inoculated seed+Zn100mg/kg, T6 = inoculated seed+ Ni100mg/kg, T7 = inoculated seed+Ni 50 mg/kg + Zn 50 mg/kg, T8 = seed+Ni 50 mg/kg + Zn 50 mg/kg.

The germination percentage of maize seeds varied between the inoculated and uninoculated seeds, as illustrated in Figure 3A. There was no significant change in seed germination% among treatments, except for T5 (inoculated seed + Zn) and T8 (Seed + Zn + Ni). The combined application of Ni and Zn (T8) hindered germination and resulted in a 40% drop in germination% when compared to the control; however, the strength of inhibition was reduced when seeds were infected with *A. terrus* (T7), and germination% increased by 33% when compared to T8. Zn substantially inhibited seed germination, with a 40% reduction in infected seeds at T5 (inoculated seed + Zn). Shoot length was affected by several treatments Figure 3B. The T5 (inoculated seed + Zn) plant had the longest shoots, which increased by 49.19% above the control. *A. tereus* (T7) considerably enhanced the shoot length in the presence of Zn and Ni, and a 22% increase in shoot length was found compared to T8. The combined application of Zn and Ni (T8) reduced shoot length. Figure 3C demonstrates that inoculation with *A. tereus* had a substantial effect on root length compared to uninoculated seeds. In the presence of Ni and Zn (T2 and T3), root length was suppressed; however, inoculation of seeds with *A. tereus* greatly enhanced root length (T5) and T6, and the % increase was 38.82 and 37.02% greater than that of T2 and T3, respectively. Similarly, the combined treatment of Ni and Zn (T8) decreased root growth, but inoculation of seeds with *A. tereus* (T7) significantly improved root length compared to T8 by 25.00%. The number of leaves varied between inoculated and uninoculated plants (Figure 3D). There was no significant difference in the number of leaves between any of the treatments, with the exception of T3 (Seed + Ni), where the increase was 10.86% and the number of leaves was much greater than that in T2 and T4. The leaf width of maize plants differed between the inoculated and uninoculated seeds, as shown in Figure 4A. Leaf width was reduced in the presence of Ni and Zn (T2 and T3); however, inoculation of seeds with *A. tereus* led to an 89% increase in leaf width compared to the control. Similarly, the combined application of Zn and Ni decreased the leaf width of T8 (Seed + Ni + Zn), while inoculation with *A. tereus* (T7) diminished the impact of Ni and Zn, resulting in a leaf width increase of 51.87% relative to T8. Inoculation with *A. tereus* showed a notable difference in plant height relative to uninoculated seeds (Figure 4B). In the presence of Ni and Zn (T2 and T3), plant height was impeded, whereas inoculation of seeds with *A. tereus* significantly increased plant height in T5 and T4, with increases 31.50 and 27.26% greater than those in T2 and T3, respectively. Similarly, the combined application of Ni and Zn (T8) decreased plant height, but inoculation of seeds with *A. tereus* (T7) tended to result in a 13.03% increase in plant height compared to T8. The inoculation of *A. tereus* had a substantial effect on leaf fresh weight compared to uninoculated seeds (Figure 4C). The leaf fresh weight was suppressed in the presence of Ni and Zn (T2 and T3), but inoculation of seeds with *A. tereus* considerably boosted the leaf fresh weight in T6 and T5, with increases of 113 and 104% more than those of T2 and T3, respectively. Likewise, the combined treatment T8 decreased leaf fresh weight, but the treatment T7 substantially raised leaf fresh weight compared to T8 by 56.41%. The results revealed a variance in dry weight in the inoculated and uninoculated treatments at 4 (d). Except for the T2 treatment, which showed a 64.83% increase in dry weight, no significant differences in dry weight were identified between the treatments. In contrast, seed inoculation with *A. tereus* increased the dry weights of T6 and T5 by 16.48 and 13.18%, respectively. Similarly, the combined treatment of Ni and Zn (T8) decreased the dry weight; however, the T7 treatment considerably increased the dry weight compared to T8 by approximately 20.87%. The results demonstrated that the inoculation of *A. tereus* significantly changed the moisture content of seeds compared to uninoculated seeds (Figure 4D). The moisture content decreased in treatments T2 and T3, whereas inoculation of seeds with *A. tereus* enhanced the moisture content in treatments T5 and T6 by 136.53 and 146.92%, respectively, compared with T2 and T3. Similarly, the T8 treatment restricted the moisture content; however, the T7 treatment enhanced the moisture content by about 68.84% compared to the T8 treatment.

### 3.4. Effect of zinc (Zn) and nickel (Ni) on maize proline protein and sugar content

**Figure 5:**
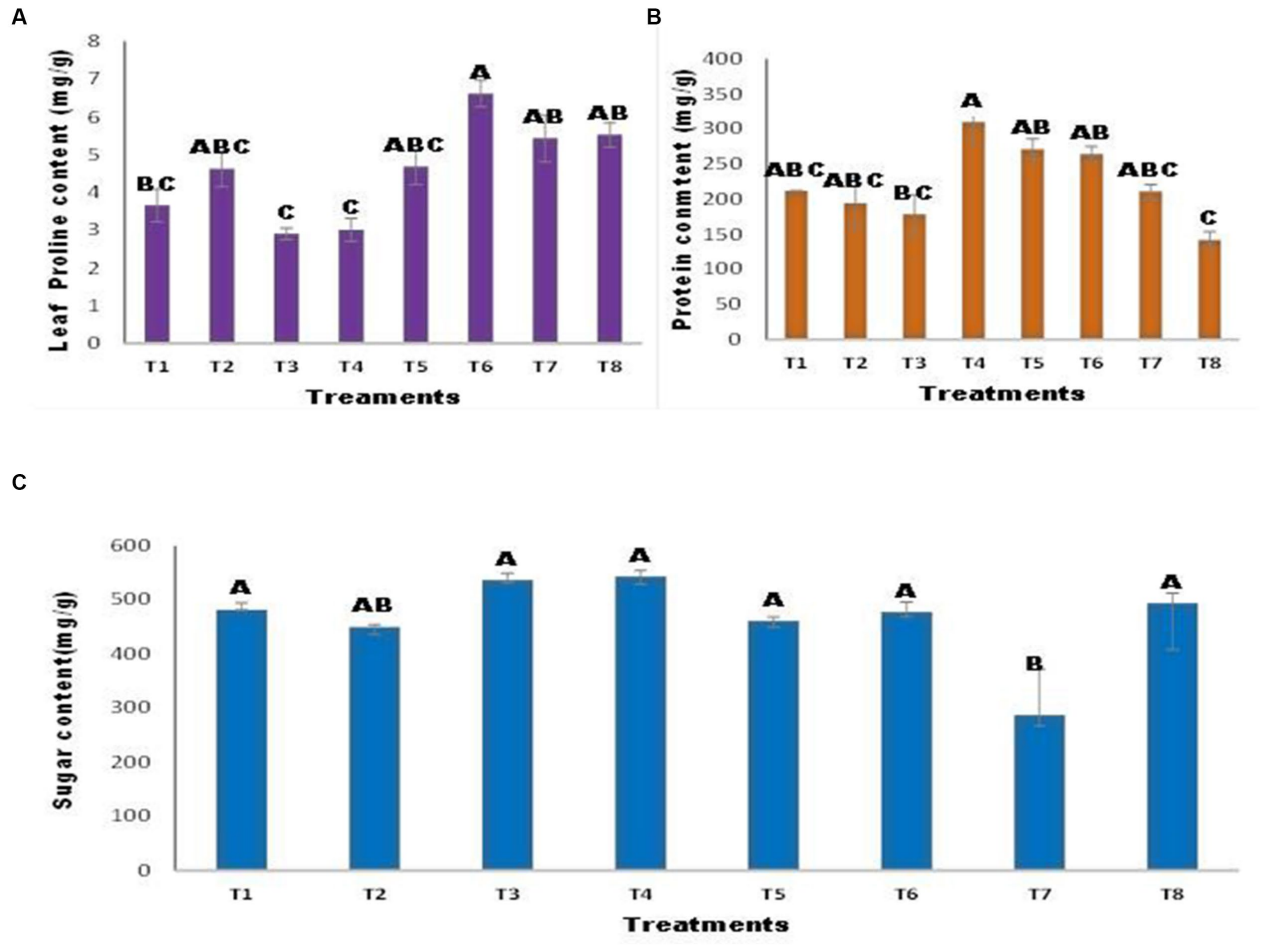
Effect of Zinc (Zn) and Nickel (Ni) on **(A)** Proline **(B)** Protein **(C)** Sugar content. The bars sharing common letter are non-significantly different otherwise vary significantly at p > 0.05. T1 = control, T2 = seed+Zn100mg/kg, T3 = seed+Ni100mg/kg, T4 = inoculated seed, T5 = inoculated seed+Zn100mg/kg, T6 = inoculated seed+ Ni100mg/kg, T7 = inoculated seed+Ni 50 mg/kg + Zn 50 mg/kg, T8 = seed+Ni 50 mg/kg + Zn 50 mg/kg.

There were variations in the proline content of the inoculated and uninoculated maize seedlings (Figure 5A). The proline content was decreased in T3 and T4; however, inoculation of seeds with *A. tereus* enhanced the proline content in T6 by approximately 80.95% compared to the control. Similarly, T8 showed a non-significant increase of 51.08% compared with T7. Protein content revealed that *A. tereus* inoculation considerably influenced protein content compared to uninoculated seeds (Figure 5B). Treatments T2 and T3 had lower protein levels. However, inoculation of seeds with *A. tereus* considerably increased the protein content of T4 and T5, with increases of 46.17 and 27.90% greater than those of T2 and T3, respectively. The results indicated that seed inoculation with *A. tereus* significantly improved the sugar content of T4 treatment plants by 12.64% compared to that of control plants (Figure 5C). *A. tereus* increased the sugar content, but Ni and Zn lowered the sugar content in T7 by 40.42% against the control.

### 3.5. Effect of zinc (Zn) and nickel (Ni) on peroxidase enzyme and superoxide dismutase enzyme

**Figure 6:**
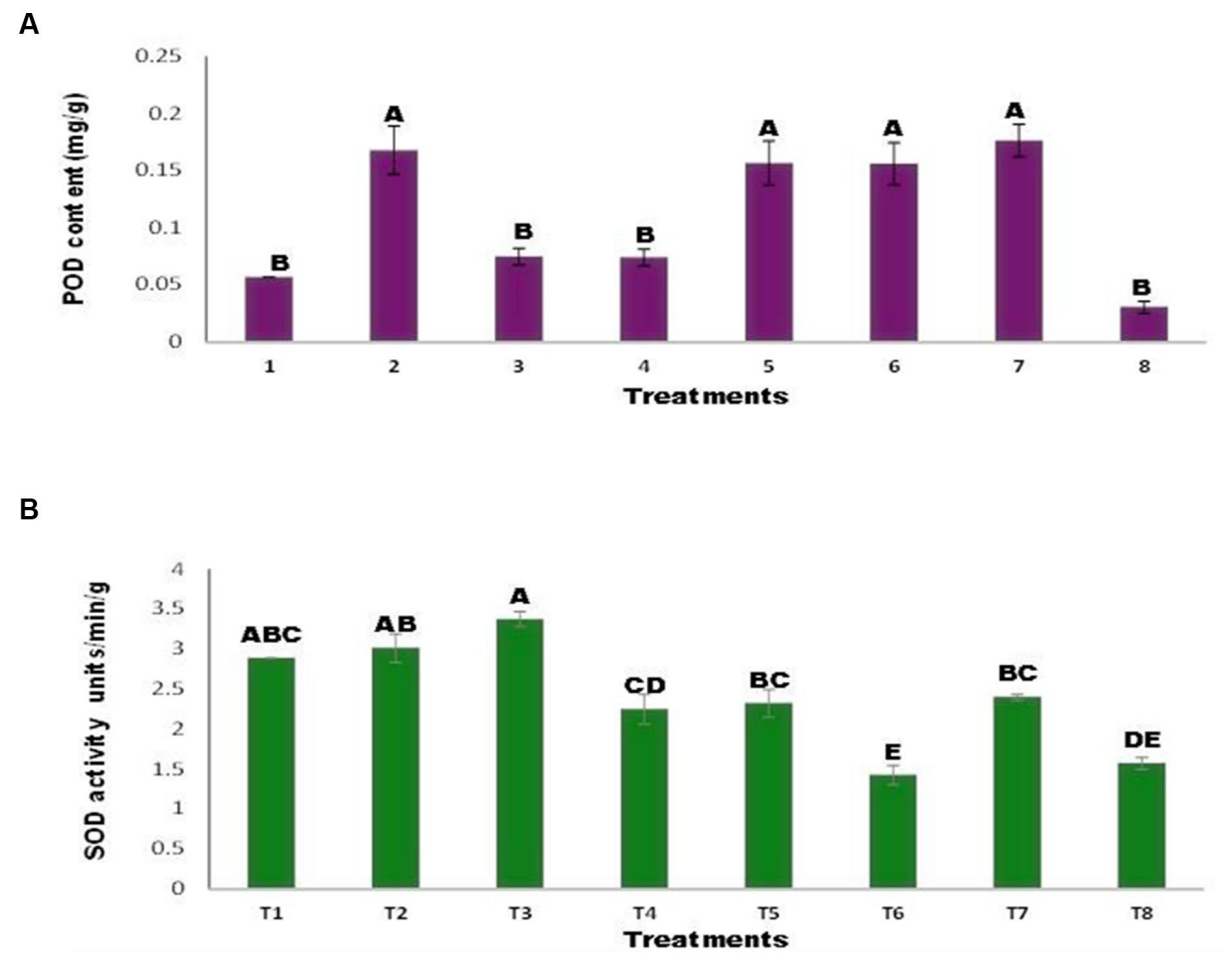
Effect of Zinc (Zn) and Nickel (Ni) on **(A)** POD **(B)** SOD. The bars sharing common letter are non-significantly different otherwise vary significantly at p > 0.05. T1 = control, T2 = seed+Zn100mg/kg, T3 = seed+Ni100mg/kg, T4 = inoculated seed, T5 = inoculated seed+Zn100mg/kg, T6 = inoculated seed+Ni100mg/kg, T7 = inoculated seed+Ni 50 mg/kg + Zn 50 mg/kg, T8 = seed+Ni 50 mg/kg + Zn 50 mg/kg.

Inoculated and uninoculated seeds varied in POD of the maize plants (Figure 6A). The POD concentrations in T1 and T8 decreased in the presence of Ni and Zn. While *Agrococus tereus* inoculation increased the POD content, T7 and a 211.47% increase over the control were observed. The results demonstrated the difference between the inoculated and uninoculated superoxide dismutase values (Figure 6B). The addition of Zn (T2, T5) resulted in insignificant changes compared with the control treatment. While the addition of Ni increased the SOD content by 16.78% (T3), inoculation of seeds with *A. tereus* in the presence of Ni (T6) decreased the SOD content by 50.68 and 57.76% relative to T3 and the control, respectively. Similarly, inoculation with *A. tereus* (T7) resulted in greater SOD content in the presence of Ni and Zn (T8), and 68.21% of the combined Ni and Zn treatments resulted in a higher SOD content than T8. In the presence of Ni, the SOD content was greater than that in all other treatments, but *A. tereus* inoculation (T6) lowered the SOD concentration by up to 50.68% relative to T3. The addition of Zn to *A. tereus* did not significantly reduce the SOD content in comparison with T2.

### 3.6. Accumulation of Zn and Ni by maize seedlings

**Figure 7:**
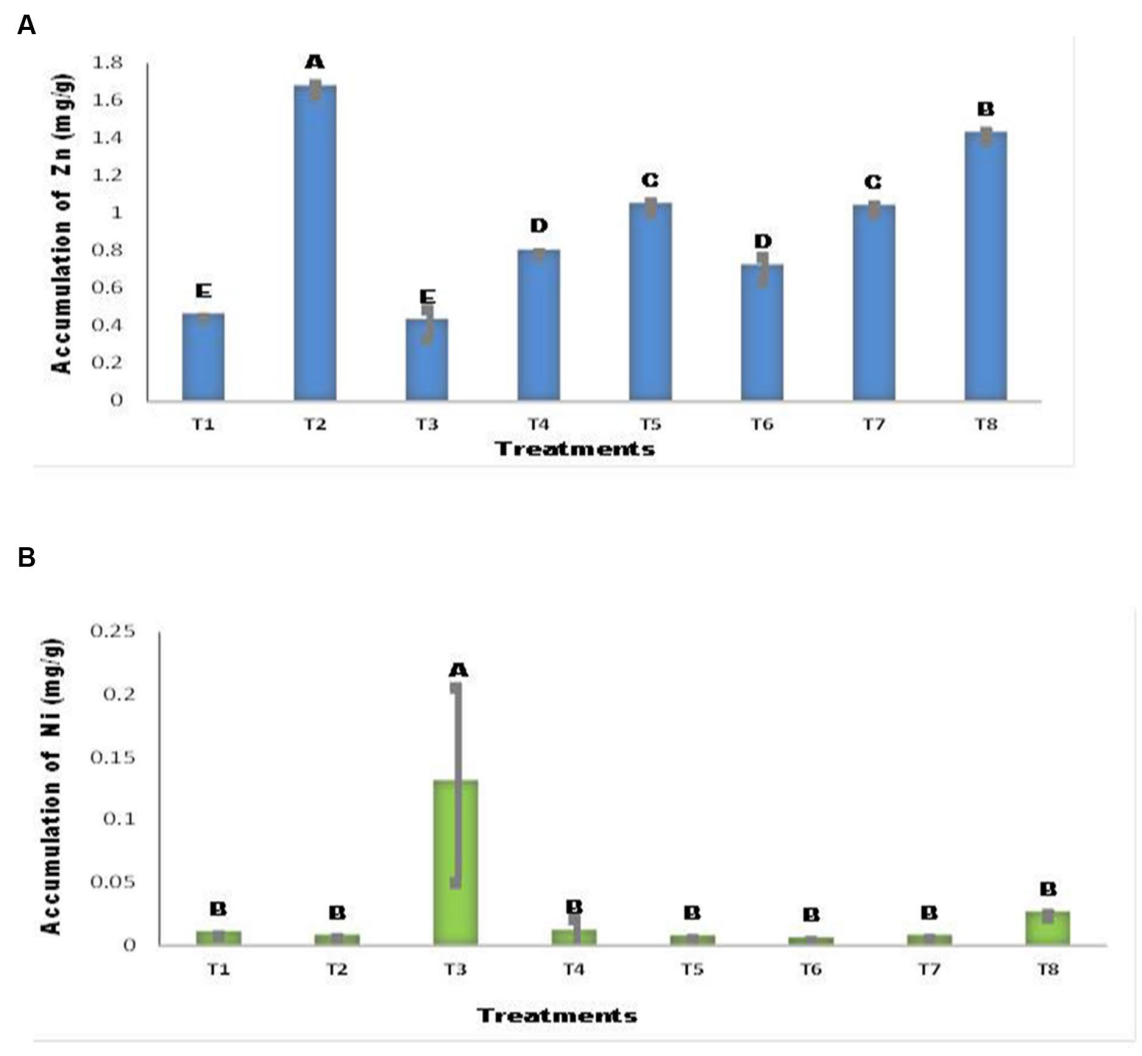
Accumulation of **(A)** Zn **(B)** Ni by Maize plant. The bars sharing common letter are non-significantly different otherwise vary significantly at *p* > 0.05. T1 = control, T2 = seed+Zn100mg/kg T3 = seed+Ni100mg/kg, T4 = inoculated seed, T5 = inoculated seed+Zn100mg/kg, T6 = inoculated seed+Ni100mg/kg, T7 = inoculated seed+Ni 50 mg/kg + Zn 50 mg/kg, T8 = seed+Ni 50 mg/kg + Zn 50 mg/kg.

The addition of Zn (T2, T5) resulted in significant changes compared with the control (Figure 7A). *A. tereus* inoculation of seeds in the presence of Ni (T6) considerably enhanced Zn accumulation in maize seedlings, with an increase of 66.80% compared with T3, whereas seeds with Ni lowered Zn content and decreased it by 6.39%. The accumulation of Ni in maize plants differed between inoculated and uninoculated seedlings (Figure 7B). The addition of Ni to the soil considerably increased the Ni content of maize seedlings (T3). This rise was tenfold greater than that of the other treatments. In all other treatments, the Ni content was not markedly different. Ni accumulation in T6 decreased by up to 43.60% with the addition of *A. tereus*.

### 3.7. Accumulation of micro and macro nutrients by maize seedlings

Accumulation of micro-and macronutrients in maize seedlings has been documented (Table 2). Copper (Cu) content varied in both inoculated and uninoculated seeds, T7 with having the highest Cu content (21.31%). The manganese (Mn) content of the inoculated seeds was greater than that of the uninoculated seeds. Maximum Mn content was found in T4 (6.27%). The inoculation of *A. tereus* increased the sodium (Na) content. T7 had the highest Na content (206.58%) and lowest Na content (16.90%). The potassium (K) content of maize seeds increased when *A. tereus* was inoculated. T7 had the highest K concentration (42.03%), whereas T2 had the lowest K concentration (35.68%). In comparison to other treatments, *A. tereus* inoculation increased the iron (Fe) content of seedlings. T7 had the highest Fe amount (48.38%), whereas T3 had the lowest (50.34%). Inoculation with *A. tereus* increased Ca content in maize seedlings. T7 had the highest calcium (Ca) content (60.85%), whereas T2 had the lowest content (27.21). Inoculation with *A. tereus* increased the amount of magnesium (Mg) in the plant, but the uninoculated plants had less Mg. T7 (92.07%) had the highest Mg level, whereas T3 had the lowest Mg content (50.76%).

**Table 2:** Accumulation of micro and macro nutrients by maize plants.

Nutrients	Nutrient concentration							
	T1	T2	T3	T4	T5	T6	T7	T8
Cu (mg/g)	0.30 ± 0.023B	0.35 ± 0.003A	0.08 ± 0.004D	0.15 ± 0.003D	0.27 ± 0.005B	0.27 ± 0.002B	0.36 ± 0.009D	0.11 ± 0.002CD
Mn (mg/g)	0.92 ± 0.004A	0.77 ± 0.03B	0.33 ± 0.001C	0.98 ± 0.0005A	0.43 ± 0.06C	0.12 ± 0.003D	0.69 ± 0.03B	0.12 ± 0.01D
Na (g/Kg)	5.77 ± 0.14D	3.81 ± 0.15E	4.03 ± 0.08E	15.20 ± 0.51B	8.99 ± 0.33C	9.14 ± 0.14C	17.70 ± 0.184A	4.79 ± 0.51DE
K (mg/g)	32.43 ± 0.49C	20.86 ± 0.52E	17.51 ± 0.30E	43.51 ± 1.117A	38.87 ± 1.00B	28.47 ± 0.61D	46.07 ± 0.78A	17.92 ± 0.56 E
Fe (mg/g)	17.36 ± 0.15C	10.65 ± 0.48D	8.62 ± 0.25C	24.40 ± 0.55AB	22.10 ± 1.05B	15.21 ± 0.39B	25.77 ± 0.58A	10.80 ± 0.17D
Ca (g/Kg)	16.43 ± 0.20B	11.96 ± 0.31D	13.81 ± 0.33D	24.48 ± 0.40A	19.59 ± 0.22B	15.92 ± 0.11C	26.43 ± 0.72A	12.66 ± 0.38D
Mg (g/Kg)	9.62 ± 0.18D	4.85 ± 0.169E	4.73 ± 0.24E	17.30 ± 0.26 BC	16.22 ± 0.55C	18.48 ± 0.51B	21.77 ± 0.13A	5.38 ± 0.51E

Treatments sharing common letter are non-significantly different otherwise vary significantly at *p* > 0.05. T1 = control, T2 = seed + Zn100mg/kg T3 = seed + Ni100mg/kg, T4 = inoculated seed, T5 = inoculated seed + Zn100mg/kg, T6 = inoculated seed + Ni100mg/kg, T7 = inoculated seed + Ni 50 mg/kg + Zn50mg/kg, T8 = seed + Ni50mg/kg + Zn50mg/kg.

## 4. Discussion

Endophytic bacteria are plant-beneficial bacteria that prevail inside plants and can facilitate plant growth under normal and stressed conditions (Lumactud and Fulthorpe, 2018) Endophytic bacteria can be beneficial to plants in several ways. Endophytic bacteria have a wide range of host (Ibáñez et al., 2017). Every plant is a host of one or more endophytes but most of the flora is unexplored. Endophytic bacteria can be isolated from these plants and used as biostimulators to develop a secure and sustainable agricultural system (Afzal et al., 2019). During the present investigation, similar findings were reported in which the isolated strain from the roots of the medicinal plant *Viburnum grandiflorum* showed variation in morphology and colony forming units (CFU). These colonies were morphologically different from one another and these results were supported by the findings of Padder et al. (2017) who demonstrated that endophytic bacterial strains with different sizes, shape textures and populations range from the roots of (*Brassica rapa L*.). In the present study, the bacterial counts were different from each other and these results are in close agreement with those of Yang et al. (2013) who demonstrated that endophytic bacteria isolated from many crops such as, cotton and sweet corn were different from each other. Jasim et al. (2014) described that this variation in the endophytic bacterial population could be due to physical, chemical, genetic, and climatic factors. Moreover, the method of isolation may also play a major role in the bacterial count. Similarly, Liaqat and Eltem (2016) isolated seven morphologically distinct bacterial strains from peach and pear roots. Approximately five out of seven strains were gram-positive, short, and rod-shaped, and two isolates were gram- and rod-shaped, demonstrating that roots are rich sources of microbial diversity. Hu et al. (2023) also reported that the abundance of bacteria in roots is due to root exudation and the digestion of a diverse class of multifarious compounds.

Janda and Abbott (2007) have reported that the use of 16S rRNA gene sequencing for identification is stunning. It is an important bioinformatics tool that provides information on genus and species identification. Several studies have shown a variety of endophytic bacteria in medicinal plants by Jalgaonwala and Mahajan (2014) and Muthukumar et al. (2017), used a similar tool (16S rRNA sequencing) and identified endophytic bacteria *Stenotrophomonas maltophilia* and *Bacillus subtilis* from the roots of the medicinal plant *Prosopis cineraria*, (Wang et al., 2016) have been identified as endophytic bacteria *Sphingomonas* and *Pseudomonas* were identified using a 16S rRNA sequencing tool from the roots of rice (*Oryza sativa* L.). El-Deeb et al. (2013) reported the presence of endophytic bacteria *Bacillus megaterium*, *Bacillus pumilus*, and *Bacillus licheniformis* from the roots of the medicinal plant *Plectranthus tenuiflorus.*

Micronutrients are needed for plant development, and their deficiency results in reduced crop output; thus, the absence of these micronutrients in staple foods such as grains may have detrimental impacts on human health (Moreno-Lora et al., 2022). Various micronutrients perform distinct functions during plant development. Ojeda-Barrios et al. (2021) found that zinc deficiency is persistent in calcareous soils and that bioavailable zinc levels are poor in almost half of the cereal-growing soils. Along with other nutrients Ni is a micronutrient required for plant metabolic activity (Tariq et al., 2022). Germination is hindered by zinc toxicity (Ozturk et al., 2006). The increased toxicity of heavy metals reduces plant biomass and growth (Lin et al., 2016). However, nickel and zinc in appropriate amounts of Ni and Zn play a crucial role in a wide range of physical processes in maize, from seed germination to yield, and without an adequate supply of these metals, plants cannot complete their life cycle. In contrast, large quantities of heavy metals can disrupt many biological processes in plants. In addition to causing toxicity in plants at high soil concentrations, Ni may also interfere with plant metabolism when combined with other minerals (Yusuf et al., 2011).

In the present study, the effects of Zn and Ni on plant height, leaf length, number of leaves, root length, fresh weight nutrient content, moisture content, and leaf width were observed, which indicated a significant decrease in germination percentage with increasing levels of zinc (Zn) and nickel (Ni) contamination. These findings are consistent with those of Jócsák et al. (2022), who observed that an increase in heavy metal content leads to a decrease in plant biomass and growth. Moreover, in the current study, seed inoculation with *A. tereus* increased plant growth. These findings were in agreement with those of Broadley et al. (2007), who stated that under normal soil conditions, plant growth improved when inoculated with PGPR due to increased carbon and nutrient content, aeration, and solubility of some essential nutrients, such as N and P (Maurya and Singh, 2010), and increased nutrient availability (Pedram, 2017). Ni and Zn inhibited root length, which is similar to the finding of Ansari et al. (2021) who found a reduction in root length in the presence of cadmium (Cd), which may have been attributable to Cd-induced damage to the protein structure. Previous studies have reported that the endophytic bacteria *Burkholderia* sp. and *Methylobacterium oryzae* enhance the growth of *Lycopersicon esculentum* under Ni and Cd stress (Madhaiyan et al., 2007). During the current investigation, similar results were observed in which the inoculation of *A. tereus* enabled the plant to overcome Ni and Zn stress and increase the root length. Weiner et al. (2012) observed that the presence of Cd hindered the growth of maize shoots. Higher concentrations of heavy metals, including Cd, Zn, and Ni, are toxic to cell membranes, causing a reduction in plant growth parameters due to cell membrane damage and the removal of ions from the damage site. Similarly, our research revealed that inoculated seeds had increased leaf length and number (Dimkpa et al., 2009; Naeem et al., 2023). Plant growth and shoot length have been reported to decrease in the presence of Ni and Zn (Sharma et al., 2020). Changes in the germination process and growth of root stems and leaves as a consequence of the adverse effects of chromium (Cr) on plant growth and development. The total dry biomass production and yield of plants were influenced by Cr exposure, and these results are in close agreement with our findings that Ni and Zn lowered the dry biomass. In the current study, however, inoculation of seeds with *A. tereus* significantly aided the plant in terms of dry weight and reduced the repercussions of heavy metals on maize plants. Ni and Zn decreased the fresh weight of maize plants, whereas *A. tereus* significantly affected the leaf fresh weight, as reported in the present study and consistent with previous findings. The inoculation of seeds with a bacterial strain in contaminated soil has the potential to increase the production of hormones in plants, which can increase their fresh weight (Iqbal et al., 2017; Zhang et al., 2023). The results of this study showed that proline content in maize plant leaves was decreased in the presence of zinc (Zn) and nickel (Ni) and these findings were comparable with those results Qiao et al. (2015), who suggested that the addition of zinc (Zn) and cadmium (Cd) to soil affected plant growth by lowering proline content. Moreover, in the current study, inoculation with *A. tereus* significantly enhanced proline content in maize plants. Similarly, Mittal et al. (2008) reported that inoculation of plants with beneficial bacteria enhanced proline activity. The increase in proline content may be due to the excretion of organic acid microbes in the soil, which helped the plant overcome metal stress. A higher amount of organic acids may be responsible for the reduction in the pH of the soil and is helpful in increasing the activity of soil enzymes, and a lower concentration of these metals could be helpful in proline activity (Shen et al., 2020). The protein content was reduced by the effects of Zn and Ni in the current study, but when seeds were inoculated with *A. tereus*, the protein content increased. These findings are supported by (Oleńska et al. (2020) and Zhao et al. (2023), who discovered that when seeds were inoculated with PGPR, there was an improvement in protein content and plant growth under normal soil conditions, which could be due to increased carbon and nutrient content, aeration, and solubility of some essential nutrients. The sugar concentration increased in rice and bean cotyledons under Cd stress (Huybrechts et al., 2019). In the current study, the sugar content was significantly reduced when plants were grown in Ni- and Zn-contaminated soil, but *A. tereus* promoted resistance in plants, and sugar content was enhanced in plants inoculated with *A. tereus*. During this study, the proline content decreased in the presence of Ni and Zn; however, *A. tereus* helped the plant withstand contaminated soil and significantly enhanced the proline content, indicating a favorable relationship between proline accumulation and plant stress. Similar outcomes have been published (Hayat et al., 2012), indicating that proline content, when subjected to stressed conditions, responded as an excellent osmolyte and conducted a diverse range of crucial functions. The current findings further demonstrate that the antioxidant enzymes SOD and POD vary considerably (Rajput et al., 2021). The variation in antioxidant enzymes was dependent on the concentration, type of heavy metal, plant species, and exposure of the plant to the contaminant in the soil. Similarly, in the current study, the presence of Ni and Zn metals in soil, even at low concentrations, had a significant effect on antioxidant enzymes, such as SOD, in maize. However, bacterial inoculation detoxifies heavy metal toxicity and reduces the SOD content in plants, which may have been caused by the action of a bacterial strain that minimized toxic effects in the soil environment for plants. Similar findings were reported by Sies et al. (2022), in which the plants under stress conditions showed reduced activity of superoxide dismutase (SOD), which may lead to a decrease in the production of glutathione (GSH) and ascorbate (ASC), resulting in a neurotic oxidative defense mechanism in plants. Dragišić Maksimović et al. (2012) found that Si can mitigate Mn toxicity in cucumber plants by increasing POD content; similarly, inoculation with *A. tereus* augmented POD content and reduced heavy metal toxicity. Microorganisms create 1-aminocyclopropane-1-carboxylate (ACC), which regulates the levels of ethylene by metabolizing it and promoting plant development when plants are under stress (Singh et al., 2011). However, according to our study, aggregation-enhanced development of maize plants may be the result of an increase in plant hormone levels caused by *A. tereus* inoculation. The production of Fe-chelating agents by microorganisms lowers the bioavailability of heavy metals. Bacteria transform the bioavailable form of Zn into an inaccessible form (Chibuike and Obiora, 2014). *A. tereus* improves the absorption of Cu, Mn, Ni, Na, Cr, Fe, Ca, Mg, and K of maize plants in comparison to uninoculated plants based on the accumulation of macro and micronutrients. Fan et al. (2021) found that A. species may increase the phytoavailability of Fe, Cd, Pb, Cr, Ni, and Cu. Siderophore complexation with metals increases metal uptake, which is why siderophores limit the phytoavailability of heavy metals and make bacterial cells more resistant to plant stress by forming complexes with metals (Ma et al., 2016). The study of the accumulation of heavy metals indicated variations in their composition, such as an increase in the concentration of Cu. Similarly, PGPR, which aided in the development of the plant shoots and roots, ultimately exhibited the same outcomes. The inoculation with *A. tereus* showed substantial potential for the absorption of micro-and macronutrients in maize plants. Additionally, previous studies have established the mechanism of PGPBs and plant pathways that contribute to the phytobiome and ultimately promote plant development. The indirect influence of micro- and macro-nutrient intake on plant growth and production has also been determined through research.

## 5. Conclusion

The current research concluded that 100 mg/kg of zinc (Zn) and nickel (Ni) was toxic for plant development; leading to a substantial reduction in plant biomass and inoculating maize seeds with *A. tereus* was efficient in lowering Zn and Ni toxicity in various plant parameters. Seed germination, shoot length, plant height, and moisture content were all significantly higher in treatment T5 (*A. tereus* + seeds + Zn 100 mg/kg) than in the uncontaminated soil. Additionally, T4 (*A. tereus* + Seeds) decreased plant defense enzymes (SOD, POD) in Ni and Zn polluted soil, but increased protein, proline, and sugar content. Moreover, *A. tereus* inoculation showed resistance against the combined stress of Ni and Zn, and boosted POD and SOD in maize. *A. tereus* inoculation aided maize plants in effectively absorbing nutrients including Cu, Mn, Ni, Na, Cr, P, Mg, K, and *Ca. A. tereus* can, thus, be suggested for future application as a possible plant growth-promoting bacterial inoculum to assist plants grown in heavy metal polluted soil and can be employed as bio-fertilizer to stimulate the plant development.

## Data availability statement

The datasets presented in this study can be found in online repositories. The names of the repository/repositories and accession number(s) can be found below: https://www.ncbi.nlm.nih.gov/genbank/, MW 979614.

## Author contributions

AsS: Formal analysis, Investigation, Software, Supervision, Writing – original draft, Writing – review & editing. AnS: Formal analysis, Investigation, Methodology, Writing – original draft. SF: Conceptualization, Data curation, Formal analysis, Investigation, Methodology, Writing – review & editing. MA: Validation, Writing – review & editing. MQ: Resources, Validation, Visualization, Writing – review & editing. UA: Writing – review & editing. Formal analysis, Investigation, Methodology, Writing – original draft. MN: Conceptualization, Software, Writing – review & editing. TB: Investigation, Methodology, Validation, Writing – review & editing. AbS: Writing – review & editing.
