# Supplementary material for: Heavy metals mitigation and growth promoting effect of endophytic Agrococcus terreus (MW 979614) in maize plants under zinc and nickel contaminated soil
Source: Front Microbiol. 2023 Nov 9;14:1255921. doi: 10.3389/fmicb.2023.1255921 (PMC10668838; doi:10.3389/fmicb.2023.1255921)
Supplement: Supplementary file 1 [file Table_1.pdf]

Table S1 Endophytic Bacterial characterization Isolated from *Viburnum grandiflorum roots*

Plant/colony	Color	Size	Margin	Texture	Gram staining	Types of bacteria	IAA	PSB	HCN
Viburnum grandiflorum (a)colony *Bacillus mycoides* MIU (Acc MW979613)	White	Small	irregular	Flate	+ve	*Bacilli*	+	+	+
Viburnum grandiflorum (b)colony	White	Large	Irregular	Flate	+ve	*Bacilli*	-	+	-
Viburnum grandiflorum (c)colony	Yellow	Small	Smooth	Flate	+ve	*Bacilli*	-	-	-
Viburnum grandiflorum (d)colony *Agrococcus terreus (*MW 979614)	Yellow	Large	Smooth	Flate	+ve	*Bacilli*	+	+	+
